# The expression of RIPK3 is associated with cell turnover of gastric mucosa in the mouse and human stomach

**DOI:** 10.1007/s10735-021-10001-5

**Published:** 2021-06-25

**Authors:** Guanglin Cui, Yaobo Yuan, Yanan Wang, Zhenfeng Li

**Affiliations:** 1 Research Group of Gastrointestinal Diseases, The Second Affiliated Hospital of Zhengzhou University, Zhengzhou, Henan China; 2 Faculty of Health Science, Nord University, Campus Levanger, Bodø, Norway; 3 Department of Surgery, The First Affiliated Hospital of Zhengzhou University, Zhengzhou, Henan China

**Keywords:** Necroptosis, RIPK3, Stomach, Mucosa

## Abstract

Necroptosis is a novel manner of programmed cell death and important for tissue development, homeostasis, damage, and repair. Activation of receptor-interacting protein kinase 3 (RIPK3), a key member of receptor-interacting protein family in contributing significantly to necroptosis, in tissues is a hallmark of cells dying by necroptosis. However, there are few studies that examine the expression of RIPK3 in the glandular cells of stomachs under physiological condition. We have therefore conducted this study to immunohistochemically characterize the key element of necroptosis, RIPK3, in the mouse and human stomach. Results showed that RIPK3 positive cells could be observed in the surface mucosal cells, granular cells, and lamina propria cells in both mouse and human stomach tissues. Ratios of PCNA/RIPK3 positive cells in the glandular cells were ~ 2.1 in mouse and ~ 4.15 in human sections respectively. Morphological and double immunofluorescence analysis confirmed that these RIPK3 positive cells were mucous, parietal and lamina propria cells. Our results indicate that the expression of RIPK3 in different cell types might contribute to cell turnover of gastric mucosa in the mouse and human stomach under physiological condition.

## Introduction

Recently, the relationship between cell death and human diseases has become a great interesting topic in research (Zhou and Yuan 2014). Traditionally, the manner of cell death can be divided into two types: necrosis and programmed cell death (PCD). Necrosis is a passive cell death and can be regarded as an extreme cell clearance method in organisms caused by pathological factors such as physics, chemistry, and pathogens. PCD is an active process of cell self-regulation, mainly characterized by genetically controlled cell-autonomous ordered death. More recently, people gradually realized that cell necrosis could also occur in an orderly manner. This new cell death form was termed as necroptosis. Studies revealed that pro-inflammatory cytokines such as tumor necrosis factor (TNF)-α play an important stimulating role in the process of necroptosis (Laster et al. 1988). TNF-α activates receptor-interacting protein kinase (RIPK) 1 (Moriwaki and Chan 2014; Silke et al. 2015; Yu, 2015), and in turn activates mixed lineage kinase domain-like (MLKL) and further regulates the downstream element RIPK3 and causes necroptosis (Sun et al. 2012; Zhang et al. 2009). Excessive necroptosis in cells will induce the release of inflammatory mediators and inflammation. Accordingly, excessive increases in necroptosis is one of the hallmarks of inflammatory diseases (Dagenais et al. 2014; Lee et al. 2020; Pierdomenico 2014), which is postulated through an disturbed intestinal epithelial cell turnover rate and homeostasis (Gunther et al. 2014, 2013), and relates to the initiation of intestinal inflammation (Dagenais et al. 2014; Gunther et al. 2013; Negroni et al. 2015; Takahashi et al. 2014; Werts, 2019). However, there are few studies to examine the expression of RIPK3 in the gastric mucosa under physiological condition.

As we have known, one of principal functions of the stomach is to create an acidic milieu that predominately depends on hydrochloric acid produced by parietal cells in the fundic glands, which is mainly controlled by the gastrin-histamine sequence (Cui and Waldum 2007). Therefore, the stomach is exposed to a high concentration of acid and various endogenous and exogenous noxious agents that could induce a rapid turnover rate in gastric cells and makes the gastric mucosa as one of the most rapidly proliferating tissues in humans. Cell proliferation and cell death are two essential parts involved in the physiological cellular turnover of gastric mucosa. Studies have shown that pathological conditions such as *Helicobacter pylori* (*H. pylori*) infection, a main cause of peptic ulcers and gastric cancer, will result in a remarkably altered cell proliferation and cell death rate in the stomach (Alzahrani et al. 2014; Chen, 1997; Cui, 2006; Jones et al. 1997; von Herbay and Rudi 2000) and contribute significantly to the development of preneoplastic and neoplastic lesions (Cui et al. 2006). Therefore, the importance of gastric cell death manner and turnover has become an attractive topic of research.

Here, we immunohistochemically characterize the key element of necroptosis, RIPK3, under physiological condition in vivo. We show a high rate of RIPK3 positive cells in mouse and human gastric specimens, RIPK3 was identified in different cell types such as surface mucous, fundic parietal and lamina propria cells in the stomachs.

## Materials and methods

### Animals

FVB/N mice: FVB/N mouse is one of the most commonly used laboratory animal models in the gastric research field worldwide. A total of 10 gastric fundic paraffin blocks of FVB/N male mice (Animal Center, Zhengzhou University, China) at the age of 6 months were included in this study. Animal study protocol was approved by the local medical ethics committee of Secondary Affiliated Hospital of Zhengzhou University (No. 182300410326).

### Human gastric specimens

A total of 20 gastric paraffin blocks taken from corpus mucosa of health human (male/female ratio: 5/15; average age: 46 years), retrieved from tissue bank at Department of Pathology, the Second Affiliated Hospital of Zhengzhou University, were included in this study. Human study protocol was approved by the institutional medical ethic review board of Zhengzhou University (No. U170410161) and written informed consent was taken from all the participants involved in the study.

### Stomach immunohistochemistry (IHC) in FVB/N male mice and human specimens

To measure gastric cell proliferation and necroptosis, RIPK3 and PCNA IHCs in the gastric specimens from male FVB/N mice and health human gastric tissues were examined in this study. Midline strips along the lesser curvature of the stomach were fixed in 10% neutral buffered formalin, processed and embedded in paraffin. Sections were cut at 4 μm, and then stained with hematoxylin and eosin (*H&E*). IHCs were performed with avidin–biotin-peroxidase complex (ABC) *Elicit* kits (Vector Laboratories, Burlingame, CA, USA) according to manufacturer’s instructions and our previous published method (Cui et al. 2017, 2018a, b, c). After antigen retrieval achieved by boiling sections 2 × 6 min, rabbit anti-RIPK3 polyclonal antibody (working dilution 1:800, Thermo Fisher Scientific, USA) and rabbit anti-PCNA monoclonal antibody (working dilution 1:100, Creative Diagnostics; Uppsala, Sweden). Primary antibodies were incubated with mouse and human sections respectively at 4 °C overnight in humidified chamber. 3-Amino-9-ethylcarbazole (*AEC*; Vector Laboratories, Burlingame, CA) was used as chromogen and slides were counterstained with Mayer’s hematoxylin (Vector Laboratories, Burlingame, CA, USA).

In addition, to illustrate active RIPK3 form was expressed in mouse gastric mucosal cells, IHC with rabbit anti-mouse phospho-RIPK3 Thr231-Ser232 monoclonal antibody (Abcam, UK) was done in mouse sections.

### Double immunofluorescence staining for RIPK3 expressed in glandular parietal and lamina propria cells

To examine the expression of RIPK3 in gastric glandular cells, double immunofluorescences with RIPK3/H^+^K^+^-ATPase subunit (to label parietal cells; working dilution 1:1000, mouse anti-hog monoclonal antibody, Affinity Bioreagents, Golden, CO, USA, for human stomach specimens; Rabbit anti- H + K + -ATPase beta/ATP4B antibody, Abcam, UK, for mouse stomach specimens respectively) in both mouse and human sections were done according to the protocol described in our previous publication (Cui et al. 2018a, 2021, 2015, 2020). After gastric fundic sections incubated with primary antibodies at 4 °C overnight, RIPK3-immunoreactivities (IRs) were developed with fluorescein isothiocyanate (FITC)-conjugated secondary antibody (Jackson ImmunoRearch Lab., West Grove, PA, USA), and H^+^K^+^-ATPase-IR was with Alexa 647-conjugated secondary antibody (Jackson ImmunoRearch Lab., West Grove, PA, USA).

To evaluate phenotypes of RIPK3-positive cells in the lamina propria, double DIFs with RIPK3/CD3 (to show RIPK3 in T lymphocytes, mouse anti-CD3 monoclonal antibody, DAKO, Carpinteria, CA, USA), RIPK3/CD34 (to show RIPK3 in CD34-positive stromal cells, mouse anti-CD34 monoclonal antibody, DAKO, Carpinteria, CA, USA) and RIPK3/SMA-alpha (to show RIPK3 in stromal myofibroblasts, mouse anti-SMA-α monoclonal antibody, DAKO, Carpinteria, CA, USA) were performed in human gastric sections. After gastric fundic sections incubated with primary antibodies at 4ºC overnight, RIP3-IR was developed with FITC-conjugated secondary antibody and CD3, CD34 and SMA-α-IRs were developed with Alexa 647-conjugated secondary antibody.

Double immunofluorescence-stained sections were observed and photographed under a confocal microscopy (LSM-700, Carl Zeiss, Jena, Germany) under 200 × magnification.

### Morphological evaluation

Only immunoreactive fundic glandular cells with appropriate morphology and location in well-oriented sections were counted under microscope. The volume densities of PCNA labeling cells and RIPK3-positive cells located in gastric glands were counted according to the published method (Zhao et al. 1998).

### Statistical analysis

The data are presented as the mean ± SEM (standard error of the mean) unless otherwise stated. *P* values were evaluated by the Mann–Whitney test. *P* values < 0.05 were considered statistically significant.

## Results

### PCNA and RIPK3-positive cells in the gastric fundic mucosa of FVB/N male mice and human

To estimate the turnover rate of glandular cells of gastric mucosa, we examined the proliferation activity labelled by PCNA and the expression of necroptosis key element RIPK3 with IHCs in mouse and human stomachs respectively.

**Fig. 1:**
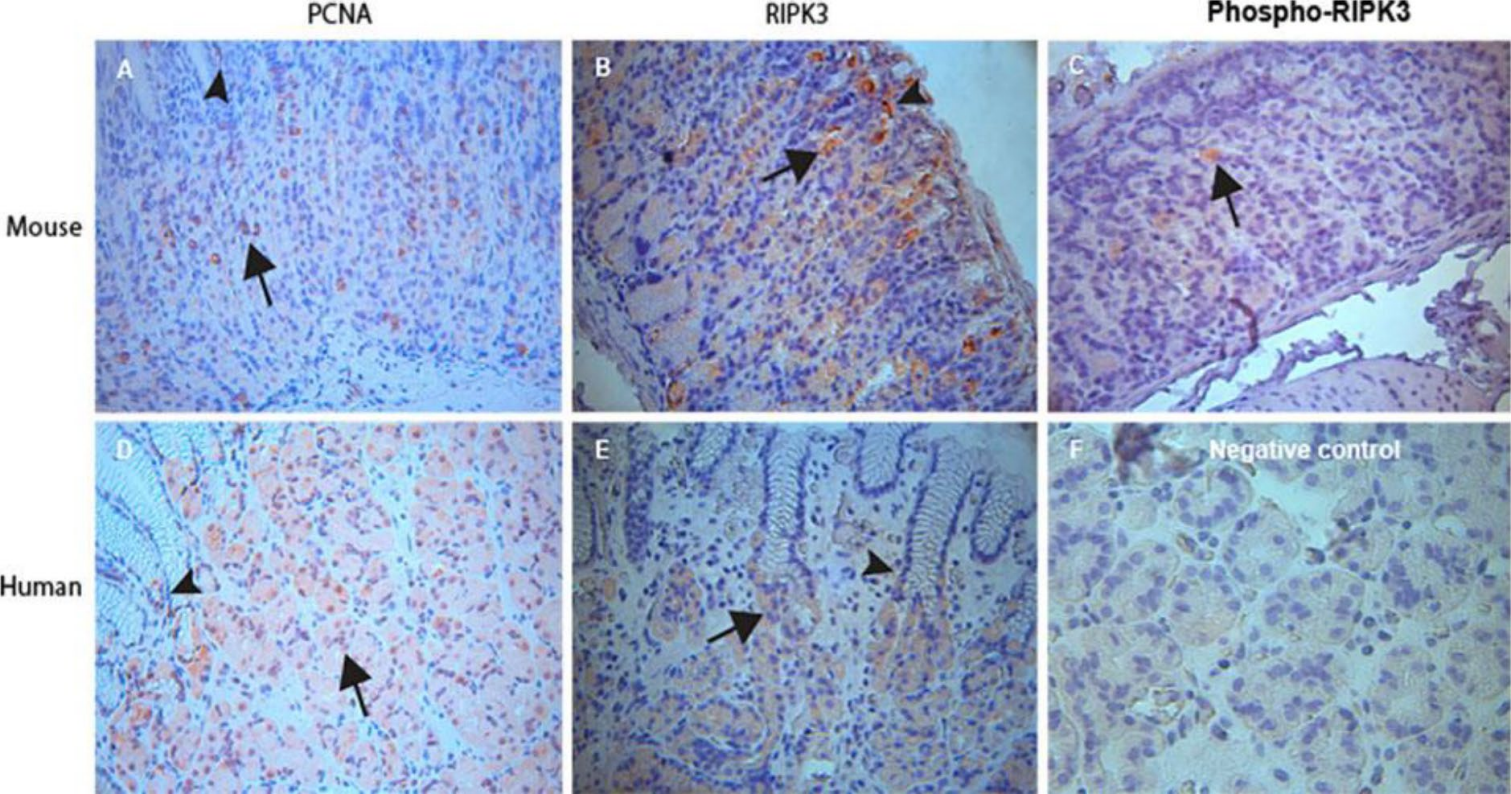
Immunohistochemical examination for proliferation rate (labelled by PCNA-positive cells), RIPK3- and Phospho-RIPK3 positive cells in the gastric fundus of FVB/N mice at an age of 6 months and gastric body of human stomach. In the stomach of FVB/N mice at the age of 6 months, image (**A**) showed a higher rate of RIPK3-positive cells on fundus glands (arrow in **A**) and some on surface mucous cells (arrowhead in **A**). **B** showed that RIPK3-positive cells were mostly located in in the fundus glands and distributed in the isthmus and neck regions (arrow in **B**), and few in the base regions. **C** showed positive cells for active form of RIPK3 (phosphor-RIPK3) in a low density in the mouse gastric glandular cells. In the body of human stomach, image (**D**) visualized that PCNA-positive cells were observed in both surface mucous cells (arrowhead in **D**) and gastric glands (arrow in **D**). RIPK3-positive cells were also shown in the same compartments of gastric body (**E**). However, RIPK3-positive cells were evenly distributed in the glands. **F** was the negative isotopic-matched control. (**A**–**F**, IHC images. Counterstained with Hematoxylin, original magnification 400 ×)

In the stomachs of FVB/N male mice, PCNA-positive cells could be observed in the surface mucous cells (arrowhead in Fig. 1A), neck cell region of the gastric fundic glands (arrow in Fig. 1A) and lamina propria cells (red arrow in Fig. 1A). Similarly, RIPK3-positive cells in the fundus of FVB/N male mice could also be detected in both the gastric mucous cells (arrowhead in Fig. 1B), gastric glandular cells (arrow in Fig. 1B) and lamina propria cells (red arrow in Fig. 1A). However, great majority of RIPK3-positive cells were within the mouse gastric glands, and predominately located in the isthmus and neck regions (Fig. 1B), and a few in the base regions. In addition, active phosphor-RIPK3-IR was shown in some human gastric glandular parietal cells (Fig. 1C).

In human stomach specimens, both PCNA positive cells (Fig. 1D) and RIPK3 positive cells (Fig. 1E) were also shown in glands (arrow), pit (surface mucous area, arrowhead) and lamina propria regions (red arrow). Many RIPK3-positive cells were observed in the fundic glands with a parietal cell morphology (Fig. 1E).

**Fig. 2:**
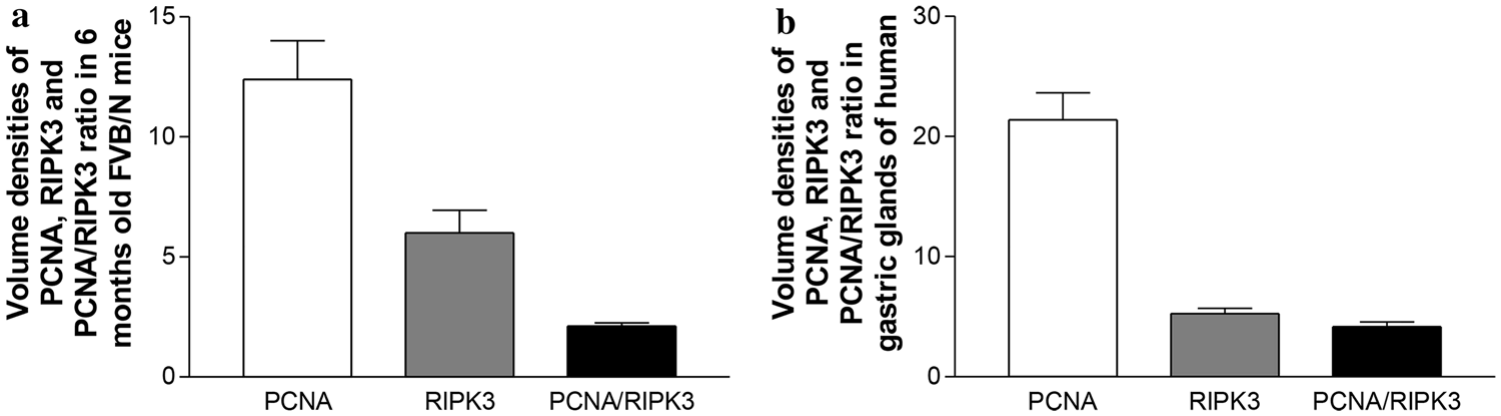
Counting data of PCNA- and RIPK3-positive cells in the fundus glands of 6-month-old FVB/N mice and body glands of human stomachs. It showed rates of PCNA-positive cell/gland (*White* bar) and RIPK3-positive cells/gland (*Grey* bar) in the fundus of mice (**A**) and the body of human stomach (**B**) respectively. The number of PCNA-positive cells/gland was higher than that of RIPK3-positive cells/gland and the PCNA/RIPK3 ratio was ~ 1.6 in the fundic glands of mice (*Black* bar in **A**), and the ratio was ~ 4.7 in the body glands of human (*Black* bar in **B**)

Since the fundic glandular cells play a key role in gastric function, we therefore counted the volume densities of PCNA- and RIPK3-positive cells and PCNA/RIPK3 ratio in fundic glands in this study. Figure 2 showed the volume densities of PCNA-positive glandular cells in both FVB/N mice (Fig. 2A) and humans (Fig. 2B). In FVB/N mice, the volume density of PCNA-positive cells (*white* bar in Fig. 2A) in fundic glands was 12.4 ± 1.6/gland. The volume density of RIPK3-positive cells in fundic glands was 6.0 ± 0.95/gland (*grey* bar in Fig. 2A). Accordingly, the ratio of PCNA/RIPK3 in the gastric fundic glands of 6-month-old FVB/N male mice was ~ 2.12 (black bar in Fig. 2A). When we divided the FVB/N mouse gastric mucosa into high and low turnover areas according to the PCNA density and counted ratio of PCNA/RIPK3 respectively, it showed that the ratio was 2,40 and 1.65/field respectively, indicating the expression of RIPK3 was higher in the area with a high proliferation rate than that in area with low proliferation rate. In human stomachs, counting data of PCNA- and RIPK3-positive cell numbers in the fundic glands revealed that volume densities of PCNA (*white* bar in Fig. 2B) and RIPK3 (*grey* bar in Fig. 2B) were (21.38 ± 2.27) and (5.25 ± 0.45) respectively. Therefore, the ratio of PCNA/RIPK3 in the human gastric fundic glands was ~ 4.15 (*black* bar in Fig. 2B).

### Phenotypic identification of RIPK3-positive cells in glandular parietal cells in both the mouse and human stomachs

The parietal cell is the main functional glandular cells that produces hydrochloric acid to kill swallowing microorganisms within the food in the stomach. To further identify some RIPK3-positive glandular cells were parietal cells, double immunofluorescence staining with H^+^K^+^-ATPase/RIPK3 antibodies were conducted in mouse and human gastric sections.

**Fig. 3:**
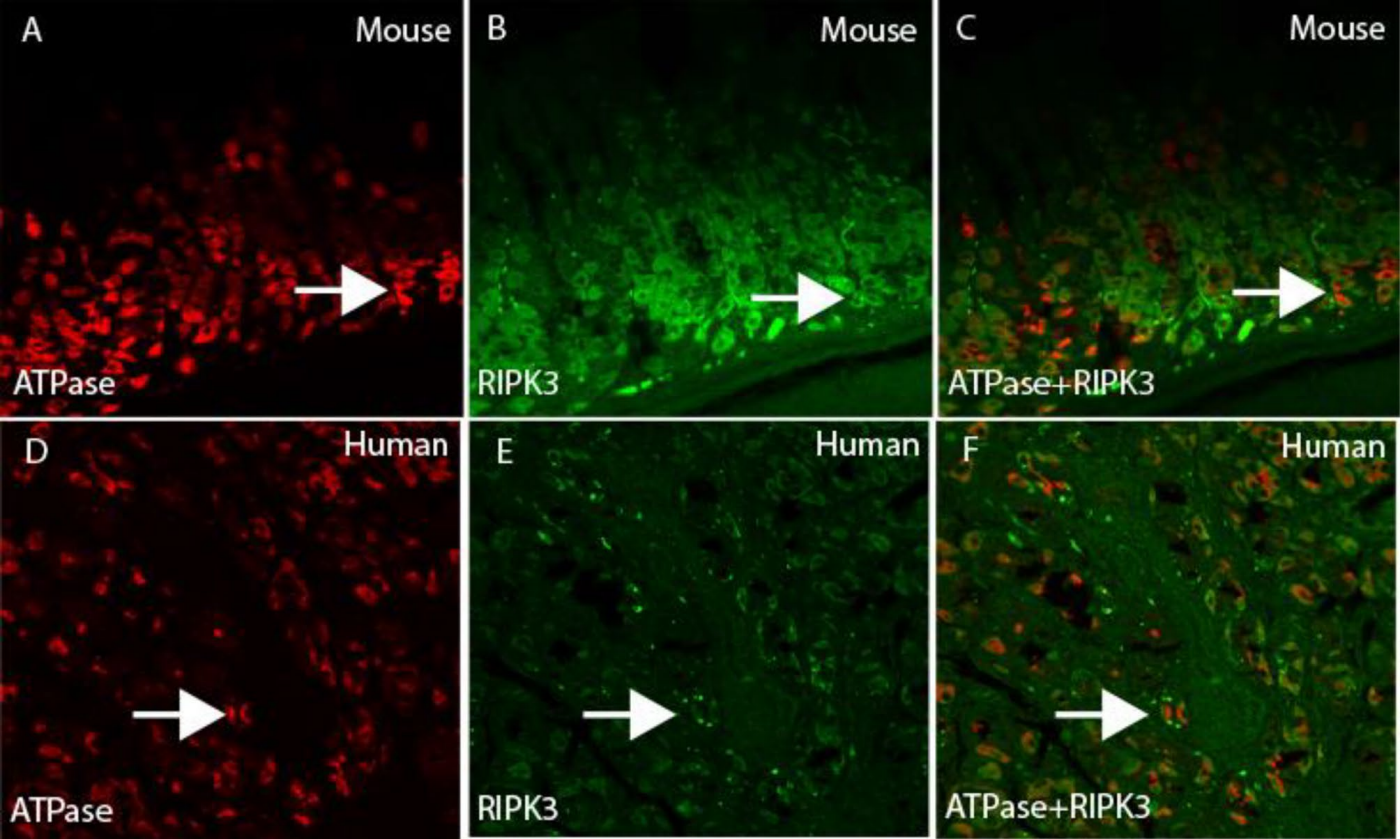
Double immunofluorescence examination on the expression of RIPK3 in glandular parietal cells in FVB/N mice and human. In the mouse gastric fundic section, RIPK3 (labelled by FITC, *green* color in **B**) was shown in H^+^K^+^-ATPase positive glandular parietal cells (labelled by Alexa-647, *red* color in **A**, **C** for merged RIPK3/H^+^K^+^-ATPase). In the human gastric body section, similar colocalization of RIPK3 (labeled by FITC, *green* color in **E**) with H^+^K^+^-ATPase-positive glandular parietal cells (labelled by Alexa-647, *red* color in **D**) was observed (**F** for merged RIPK3/H^+^K^+^-ATPase images). (**A**–**F**, double immunofluorescence staining, original magnification 200×). (Color figure online)

Double immunofluorescence images clearly demonstrated that some of RIPK3-positive glandular cells were also positive for H^+^K^+^-ATPase (Fig. 3A–C for mouse gastric section and Fig. 3D–F for human gastric section).

### Phenotypic identification of RIPK3-positive cells in the lamina propria of human stomachs

**Fig. 4:**
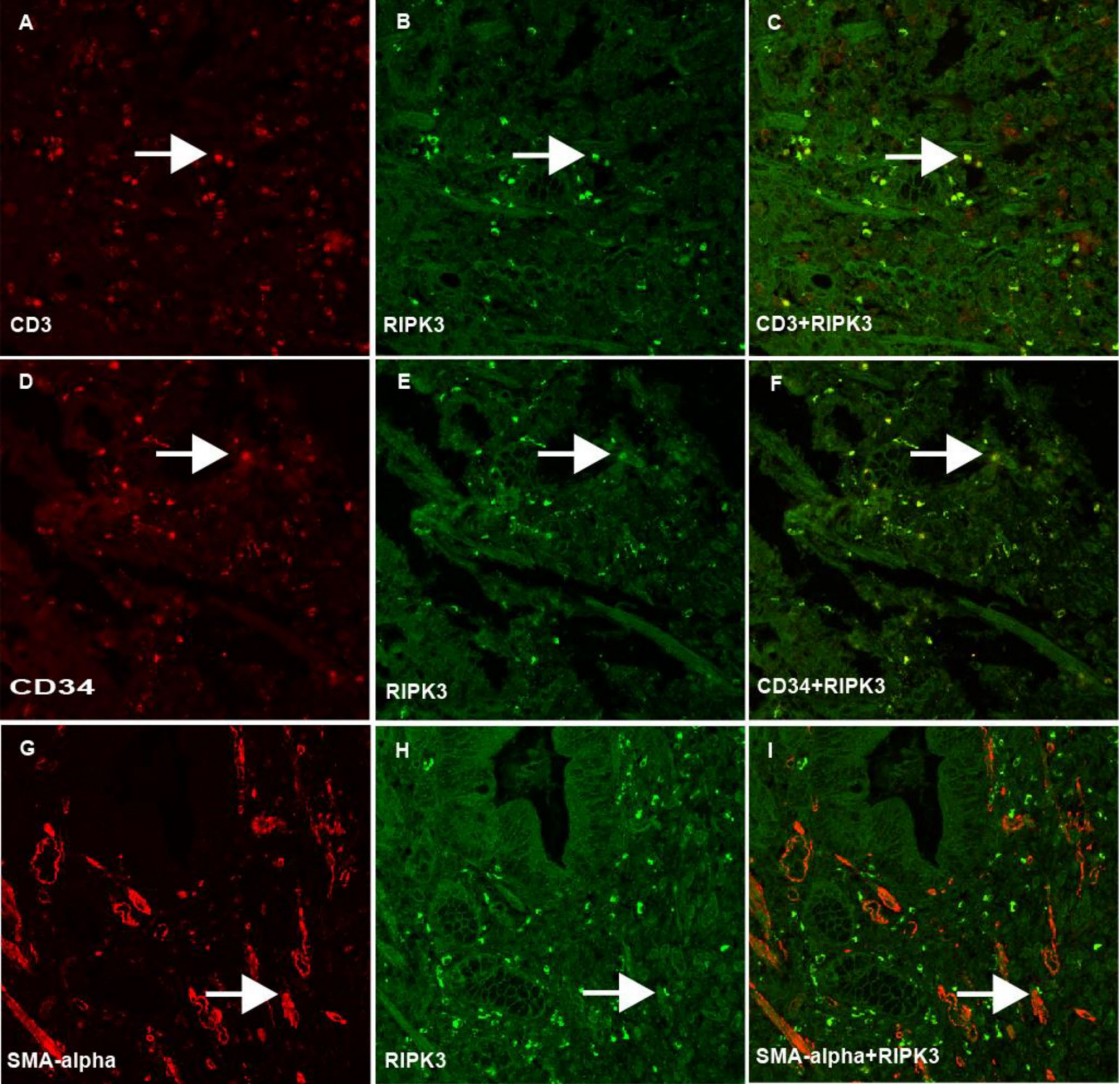
Double immunofluorescence examination of RIPK3-positive cell phenotypes in human gastric lamina propria. Phenotypic analysis revealed that RIPK3-positive cells (**B**, **E** & **H**) located in the lamina propria of human stomach were CD3-positive (**A**) lymphocytes (merged image, **C**), CD34-positive (**D**) stromal cells (merged image, **F**) and SMA-α-positive (**G**) stromal cells (merged image, **I**). (**A**–**L**, double immunofluorescence-stained confocal images, original magnification 200×)

To examine the phenotypes of RIPK3 in the lamina propria of human stomachs, we performed an additional double immunofluorescence staining with different antibodies and were able to show that phenotypes of RIPK3-positive cells in the lamina propria were CD3-positive lymphocytes, CD34-positive stromal cells and SMA-alpha-positive stromal myofibroblasts in human gastric specimens (see Fig. 4).

## Discussion

Under the physiological condition, the population of gastric mucosal cells is maintained by the balance between cell loss and self-renewal. Disruption of this homeostasis will result in significant pathological changes of the stomach. Activation of RIPK3 in tissues is a hallmark of cells dying by necroptosis (Tonnus, 2019; Webster et al. 2018). In this study, we were able to show the densities of PCNA/RIPK3 positive cells in the gastric glandular cells of mice and human, providing a basic information regarding the proliferation/necroptosis in the gastric glandular cells. Our results (Figs. 1 and 2) clearly shown visible RIPK3 positive cells and a ratio of PCNA/RIPK3 positive cells in the gastric glandular cells, suggesting a necroptotic turnover rate in both the mouse and human glandular cells. Therefore, current studies of gastric glandular cell turnover rate may provide important information to understand gastric glandular cell homeostasis under physiological condition (Barker et al. 2010; Willet and Mills 2016; Ye et al. 2018). For example, cell death is an essential process for tissue development and homeostasis in human body as a way of eliminating infected, damaged or aged cells (Gunther et al. 2014), excessive cell loss by enhanced glandular cell death will induce atrophy or ulceration while excessive proliferation or prolonged cell life span can lead to hyperplasia (Jones et al. 1999).

In this study, we have observed RIPK3 immunoreactivity in different types of gastric cells e.g. surface mucous cells, glandular cells, and lamina propria cells in both mouse and human stomach. Interestingly, we also showed that active phospho-RIPK3 was expressed in glandular cells at a low density. This observation indicates that some of RIPK3 could further go into the active form and finally induce glandular cell death. When we counted the ratio of PCNA/RIPK3 in high and low turnover gastric glandular regions respectively in the FVB/N mice, it showed that the expression of RIPK3 was higher in the area with a high proliferation rate than that in area with low proliferation rate. This finding suggested that the cell death might be elevated according to rate of proliferation, which could be important for the maintenance of an balanced cell turnover in the gastric mucosa. Because the parietal cell is the main gastric glandular cell and its main physiological function is to produce hydrochloric acid that can kill swallowed microorganisms within the food in the stomach. Therefore, to keep a balance of death/proliferation of parietal cells is critical for cellular and functional homeostasis. While the disrupted turnover of parietal cells under pathological conditions such as chronic *H. pylori* infection might result in an excessive parietal cell loss, which is a well-known glandular atrophic process and gastric cancer precursor, will significantly increase the risk for gastric cancer, Wang et al. have found that a high positive rate of RIPK3 in different types of gastric cells including the mucosal epithelial cells, smooth muscle cells and fibroblasts, while a negative for glandular epithelium in the stomach of mice (Wang et al. 2016). Such different findings could be resulted from the mice strains used in the studies. In their study, SPF BALB/c mice were used (Wang et al. 2016), and in our study FVB mice were used for the identification of necroptotic gastric cells. The RIPK3-positive cells located in the surface mucous cells can be easily identified by their location and morphology. To analyze the phenotypes of RIPK3 in other gastric cells such as glandular parietal cells, lamina propria cells, double immunofluorescence staining with different specific antibodies was conducted. We were able to show that RIPK3-positive glandular cells were mostly H + K + -ATPase positive parietal cells in both mouse and human. In lamina propria, RIPK3 was expressed by several types of lamina propria cells including CD3-positive lymphocytes, CD34-positive stromal cells and SMA-alpha-positive stromal myofibroblasts in human gastric specimens (see Fig. 4). These findings suggested that necroptosis could be a common cell death manner and occurred in different cell types in the human stomachs. Finally, we have validated that some of the RIPK3-positive glandular parietal cells were also positive for phosphorylated RIPK3, indicating an activation of RIPK3 in parietal cells.

The counting data showed that the ration of PCNA/RIPK3 positive cells/gland in human stomach is approximately 4.15, which was slightly higher than that the ratio (2.12/gland) in mice. This finding might suggest that the turnover rate of glandular cells in human stomach is slightly higher than that in mouse stomach. It  was a surprise to find that the exact value of cell death in human or mouse gastric glands or cells were unavailable when we searched literature. So, it is impossible to compare our data with available publications. In addition, cell death might have several forms including necrosis in response pathological factors such as physics, chemistry, and pathogens and PCD. Necrosis usually occurs in response to pathological factors such as physics, chemistry, and pathogens; whereas PCD mainly including apoptosis and necroptosis is an active process of cell self-regulation and characterized by genetically controlled cell-autonomous ordered death. Interestingly, when researchers studied PCD in the gastric glandular cells, elevated elements related to PCD e.g. caspase-3 and caspse-8 under pathological conditions are frequently found (Bockerstett et al. 2018; Cui et al. 2006; Le’Negrate et al. 2001). However, PCD cells with significant morphological changes are rarely observed in various tissues under physiological circumstances (Poon et al. 2014). Indeed, our results of PCNA/necrosis ratio in mouse sections showed that it is ~ 0.032/field, which is much lower than the ratio of PCNA/RIPK3. The possible reasons for such a low rate of PCNA/necrosis in the normal stomach might be: 1. The removal rate of cell undergoing PCD is very high under physiological conditions and dying cells are quickly removed either by tissue-resident professional or neighboring non-professional phagocytes and immature dendritic cells through a coordinated manner of multiple steps (Poon et al. 2014; Ravichandran 2010); 2. Not all the cells with high RIPK3 expression will develop into active form (RIPK3 → phosphorylated RIPK3) that could induce the process of necroptosis finally. These reasons make the accurate measurement of PCD rate in gastric sections by counting dying cells with morphological changes in histological sections very difficult under physiological circumstances (Poon et al. 2014; Ravichandran 2010).

Recently, Karam has summarized the literature regarding the renewal capacity of parietal cells in mice (Karam 2010). Their review article indicates that old parietal cells will undergo degeneration and elimination after an average lifespan of about 54 days, and new parietal cells will be developed directly from the stem-like pre-parietal cells through a mature process (Karam 2010). The homeostasis of parietal cells is kept by the balanced rate of renewal and death. Revealing the dynamic features of parietal cell renewal/death can help in a better understanding of the structural integrity and physiological functions of the gastric glands. For example, when the acid secretion amount should be at a normal amount when the death/proliferation of parietal cells is normal, because the population of parietal cells will be at a certain stable level. On the contrary, the excessive death of parietal cells can lead to a reduced density of parietal cells, and further result in a decreased acid secretion function. Clinically, long-term over-suppression of parietal cell acid secretion function by administration of proton pump inhibitors could cause an overgrowth of bacterial in the intestine. Thus, our current results may have a particular clinical significance that increase of necroptosis is involved in the process of acid-secreting parietal cell atrophy under pathological conditions such as *H. pylori* infection. Previous studies have demonstrated that enhanced gastric epithelial cell apoptosis, as one of the major cell PCD manners, is observed during *H. pylori* infection (Chen et al. 1997; Jones et al. 1997), which is significantly associated with the development of chronic gastritis, peptic ulcers, and gastric carcinogenesis (Rogers et al. 2005; Targa et al. 2007; Xia and Talley 2001). We have demonstrated that chronic *Helicobacter* infection and induced hypergastrinemia could lead to increased apoptosis in glandular parietal cells and contribute to the development of gastric cancer in *Helicobacter* infected mice (Cui, 2004; Cui et al. 2006). Burclaff and colleagues (Burclaff et al. 2017) have developed a mouse model that expresses the diphtheria toxin receptor specifically in parietal cells to induce their death and found this to increase proliferation in the normal stem cell zone and neck area. In addition, several proinflammatory cytokines such as TNF-α and interleukin (IL)-17A have been shown to promote the parietal cell atrophy and metaplasia in isolated parietal cells or mouse models (Bockerstett et al. 2018; Neu, 2003), whether the induction of atrophy is associated with the necroptosis induced by these cytokines would be an interesting future topic. Furthermore, it is postulated that necroptosis, as a novel PCD manner, may be involved in the gastric cell death induced by *H. pylori* infection. Indeed, Radin et al. have demonstrated that *Helicobacter* toxin Vac A could induce necroptosis in epithelial cells in vitro (Radin et al. 2011). Therefore, it is worth studying in the future the role of necroptosis in chronic *H. pylori* infection-induced parietal cell atrophy.

In conclusion, our present study has revealed that a high rate of RIPK3 expression in different cell types, particularly in glandular cells, might contribute to cell turnover of gastric mucosa in the mouse and human stomach under physiological condition.

## Data Availability

Data to support this study were based on analysis of immunohistochemistry and histological diagnosis from mice and human subjects admitted to our hospital.

## Abbreviations

ABC: Avidin–Biotin-Peroxidase Complex
FITC: Fluorescein Isothiocyanate
*H. pylori*: *Helicobacter pylori*
IHC: Immunohistochemistry
IL: Interleukin
MLKL: Mixed Lineage Kinase Domain-Like
PCD: Programmed Cell Death
RIPK: The Receptor-Interacting Protein Kinase
TNF: Tumor Necrosis Factor

## References

[CR1] Alzahrani S, Lina TT, Gonzalez J, Pinchuk IV, Beswick EJ, Reyes VE (2014). Effect of Helicobacter pylori on gastric epithelial cells. World J Gastroenterol.

[CR2] Barker N (2010). Lgr5(+ve) stem cells drive self-renewal in the stomach and build long-lived gastric units in vitro. Cell Stem Cell.

[CR3] Bockerstett KA (2018). Interleukin-17A promotes parietal cell atrophy by inducing apoptosis. Cell Mol Gastroenterol Hepatol.

[CR4] Burclaff J, Osaki LH, Liu D, Goldenring JR, Mills JC (2017). Targeted apoptosis of parietal cells is insufficient to induce metaplasia in stomach. Gastroenterology.

[CR5] Chen G (1997). Apoptosis in gastric epithelial cells is induced by *Helicobacter pylori* and accompanied by increased expression of BAK. Biochem Biophys Res Commun.

[CR6] Cui G, Waldum HL (2007). Physiological and clinical significance of enterochromaffin-like cell activation in the regulation of gastric acid secretion. World J Gastroenterol.

[CR7] Cui G (2004). Overexpression of glycine-extended gastrin inhibits parietal cell loss and atrophy in the mouse stomach. Cancer Res.

[CR8] Cui G (2006). Gastrin-induced apoptosis contributes to carcinogenesis in the stomach. Lab Invest.

[CR9] Cui G (2015). Dynamics of the IL-33/ST2 network in the progression of human colorectal adenoma to sporadic colorectal cancer. Cancer Immunol Immunother.

[CR10] Cui G, Yuan A, Zhu L, Florholmen J, Goll R (2017). Increased expression of interleukin-21 along colorectal adenoma-carcinoma sequence and its predicating significance in patients with sporadic colorectal cancer. Clin Immunol.

[CR11] Cui G (2018). Tumor-associated fibroblasts and microvessels contribute to the expression of immunosuppressive factor indoleamine 2, 3-dioxygenase in human esophageal cancers. Pathol Oncol Res.

[CR12] Cui G, Ren J, Xu G, Li Z, Zheng W, Yuan A (2018). Cellular and clinicopathological features of the IL-33/ST2 axis in human esophageal squamous cell carcinomas. Cancer Cell Int.

[CR13] Cui G, Yuan A, Sun Z, Zheng W, Pang Z (2018). IL-1beta/IL-6 network in the tumor microenvironment of human colorectal cancer. Pathol Res Pract.

[CR14] Cui G, Yuan A, Li Z, Goll R, Florholmen J (2020). ST2 and regulatory T cells in the colorectal adenoma/carcinoma microenvironment: implications for diseases progression and prognosis. Sci Rep.

[CR15] Cui G, Li Z, Florholmen J, Goll R (2021). Dynamic stromal cellular reaction throughout human colorectal adenoma-carcinoma sequence: a role of TH17/IL-17A. Biomed Pharmacother.

[CR16] Dagenais M, Douglas T, Saleh M (2014). Role of programmed necrosis and cell death in intestinal inflammation. Curr Opin Gastroenterol.

[CR17] Gunther C, Neumann H, Neurath MF, Becker C (2013). Apoptosis, necrosis and necroptosis: cell death regulation in the intestinal epithelium. Gut.

[CR18] Gunther C, Buchen B, Neurath MF, Becker C (2014). Regulation and pathophysiological role of epithelial turnover in the gut. Semin Cell Dev Biol.

[CR19] Jones NL, Shannon PT, Cutz E, Yeger H, Sherman PM (1997). Increase in proliferation and apoptosis of gastric epithelial cells early in the natural history of *Helicobacter pylori* infection. Am J Pathol.

[CR20] Jones MK, Tomikawa M, Mohajer B, Tarnawski AS (1999). Gastrointestinal mucosal regeneration: role of growth factors. Front Biosci.

[CR21] Karam SM (2010). A focus on parietal cells as a renewing cell population. World J Gastroenterol.

[CR22] Laster SM, Wood JG, Gooding LR (1988). Tumor necrosis factor can induce both apoptic and necrotic forms of cell lysis. J Immunol.

[CR23] Le’Negrate G, Ricci V, Hofman V, Mograbi B, Hofman P, Rossi B (2001). Epithelial intestinal cell apoptosis induced by Helicobacter pylori depends on expression of the cag pathogenicity island phenotype. Infect Immun.

[CR24] Lee SH (2020). Inhibition of RIPK3 pathway attenuates intestinal inflammation and cell death of inflammatory bowel disease and suppresses necroptosis in peripheral mononuclear cells of ulcerative colitis patients. Immune Netw.

[CR25] Moriwaki K, Chan FK (2014). Necrosis-dependent and independent signaling of the RIP kinases in inflammation. Cytokine Growth Factor Rev.

[CR26] Negroni A, Cucchiara S, Stronati L (2015). Apoptosis, necrosis, and necroptosis in the gut and intestinal homeostasis. Mediators Inflamm.

[CR27] Neu B (2003). TNF-alpha induces apoptosis of parietal cells. Biochem Pharmacol.

[CR28] Pierdomenico M (2014). Necroptosis is active in children with inflammatory bowel disease and contributes to heighten intestinal inflammation. Am J Gastroenterol.

[CR29] Poon IK, Lucas CD, Rossi AG, Ravichandran KS (2014). Apoptotic cell clearance: basic biology and therapeutic potential. Nat Rev Immunol.

[CR30] Radin JN, Gonzalez-Rivera C, Ivie SE, McClain MS, Cover TL (2011). Helicobacter pylori VacA induces programmed necrosis in gastric epithelial cells. Infect Immun.

[CR31] Ravichandran KS (2010). Find-me and eat-me signals in apoptotic cell clearance: progress and conundrums. J Exp Med.

[CR32] Rogers AB, Taylor NS, Whary MT, Stefanich ED, Wang TC, Fox JG (2005). Helicobacter pylori but not high salt induces gastric intraepithelial neoplasia in B6129 mice. Cancer Res.

[CR33] Silke J, Rickard JA, Gerlic M (2015). The diverse role of RIP kinases in necroptosis and inflammation. Nat Immunol.

[CR34] Sun L (2012). Mixed lineage kinase domain-like protein mediates necrosis signaling downstream of RIP3 kinase. Cell.

[CR35] Takahashi N (2014). RIPK1 ensures intestinal homeostasis by protecting the epithelium against apoptosis. Nature.

[CR36] Targa AC, Cesar AC, Cury PM, Silva AE (2007). Apoptosis in different gastric lesions and gastric cancer: relationship with *Helicobacter pylori*, overexpression of p53 and aneuploidy. Genet Mol Res.

[CR37] Tonnus W (2019). The pathological features of regulated necrosis. J Pathol.

[CR38] von Herbay A, Rudi J (2000). Role of apoptosis in gastric epithelial turnover. Microsc Res Tech.

[CR39] Wang Q, Yu M, Zhang K, Liu J, Tao P, Ge S, Ning Z (2016). Expression profile and tissue-specific distribution of the receptor-interacting protein 3 in BALB/c mice. Biochem Genet.

[CR40] Webster JD, Solon M, Haller S, Newton K (2018). Detection of necroptosis by phospho-RIPK3 immunohistochemical labeling methods. Mol Biol.

[CR41] Werts AD (2019). A Novel Role for Necroptosis in the Pathogenesis of Necrotizing Enterocolitis. Cell Mol Gastroenterol Hepatol.

[CR42] Willet SG, Mills JC (2016). Stomach organ and cell lineage differentiation: from embryogenesis to adult homeostasis. Cell Mol Gastroenterol Hepatol.

[CR43] Xia HH, Talley NJ (2001). Apoptosis in gastric epithelium induced by *Helicobacter pylori* infection: implications in gastric carcinogenesis. Am J Gastroenterol.

[CR44] Ye W (2018). Regulation of gastric Lgr5+ve cell homeostasis by bone morphogenetic protein (BMP) signaling and inflammatory stimuli. Cell Mol Gastroenterol Hepatol.

[CR45] Yu X (2015). Neoalbaconol induces cell death through necroptosis by regulating RIPK-dependent autocrine TNFalpha and ROS production. Oncotarget.

[CR46] Zhang DW (2009). RIP3, an energy metabolism regulator that switches TNF-induced cell death from apoptosis to necrosis. Science.

[CR47] Zhao CM, Chen D, Andersson K, Hakanson R (1998). ECL cells of the rat stomach: development of lipofuscin in response to sustained gastrin stimulation. Cell Tissue Res.

[CR48] Zhou W, Yuan J (2014). Necroptosis in health and diseases. Semin Cell Dev Biol.

